# The mechanism by which a distinguishing arabinofuranosidase can cope with internal di-substitutions in arabinoxylans

**DOI:** 10.1186/s13068-018-1212-y

**Published:** 2018-08-11

**Authors:** Camila Ramos dos Santos, Priscila Oliveira de Giuseppe, Flávio Henrique Moreira de Souza, Letícia Maria Zanphorlin, Mariane Noronha Domingues, Renan Augusto Siqueira Pirolla, Rodrigo Vargas Honorato, Celisa Caldana Costa Tonoli, Mariana Abrahão Bueno de Morais, Vanesa Peixoto de Matos Martins, Lucas Miranda Fonseca, Fernanda Büchli, Paulo Sergio Lopes de Oliveira, Fábio Cesar Gozzo, Mário Tyago Murakami

**Affiliations:** 1 Brazilian Bioethanol Science and Technology Laboratory (CTBE), Brazilian Center for Research in Energy and Materials (CNPEM), Campinas, Sao Paulo 13083-970 Brazil; 2 Brazilian Biosciences National Laboratory (LNBio), Brazilian Center for Research in Energy and Materials (CNPEM), Campinas, Sao Paulo 13083-970 Brazil; 3 Dalton Mass Spectrometry Laboratory, Institute of Chemistry, University of Campinas, Campinas, Sao Paulo 13083-861 Brazil

**Keywords:** Glycoside hydrolase family 51, Arabinofuranosidase, Arabinoxylan, Molecular mechanism, Oligomerization, Crystal structure

## Abstract

**Background:**

Arabinoxylan is an abundant polysaccharide in industrially relevant biomasses such as sugarcane, corn stover and grasses. However, the arabinofuranosyl di-substitutions that decorate the xylan backbone are recalcitrant to most known arabinofuranosidases (Abfs).

**Results:**

In this work, we identified a novel GH51 Abf (*Xac*Abf51) that forms trimers in solution and can cope efficiently with both mono- and di-substitutions at terminal or internal xylopyranosyl units of arabinoxylan. Using mass spectrometry, the kinetic parameters of the hydrolysis of 3^3^-α-l-arabinofuranosyl-xylotetraose and 2^3^,3^3^-di-α-l-arabinofuranosyl-xylotetraose by *Xac*Abf51 were determined, demonstrating the capacity of this enzyme to cleave arabinofuranosyl linkages of internal mono- and di-substituted xylopyranosyl units. Complementation studies of fungal enzyme cocktails with *Xac*Abf51 revealed an increase of up to 20% in the release of reducing sugars from pretreated sugarcane bagasse, showing the biotechnological potential of a generalist GH51 in biomass saccharification. To elucidate the structural basis for the recognition of internal di-substitutions, the crystal structure of *Xac*Abf51 was determined unveiling the existence of a pocket strategically arranged near to the − 1 subsite that can accommodate a second arabinofuranosyl decoration, a feature not described for any other GH51 Abf structurally characterized so far.

**Conclusions:**

In summary, this study reports the first kinetic characterization of internal di-substitution release by a GH51 Abf, provides the structural basis for this activity and reveals a promising candidate for industrial processes involving plant cell wall depolymerization.

## Background

Arabinoxylan is a hemicellulosic polysaccharide composed of a β-1,4-linked xylose backbone, which can be mono-substituted (at O-3) or di-substituted (at O-2 and O-3) with α-L-arabinofuranosyl residues (Ara*f*) and eventually with (4-*O*-methyl) glucuronic acid [1, 2]. Industrially relevant biomasses such as sugarcane [3], corn stover [4] and grasses are rich in arabinoxylans, which can represent up to 50% (w w^−1^) of their polysaccharides in the secondary wall [2]. Moreover, arabinoxylans from cereals stimulate the activity of beneficial bacteria in the colon of humans and animals, being considered a source of prebiotic oligosaccharides with promising health-promoting properties [5, 6].

To consume arabinoxylans, the microorganisms produce a set of glycoside hydrolases including α-l-arabinofuranosidases (EC 3.2.1.55) to release the Ara*f* decorations, endo-β-1,4-xylanases (EC 3.2.1.8), which depolymerize the backbone, and β-xylosidases (EC 3.2.1.37) to convert xylooligosaccharides into xylose. Xylanases are mainly categorized into families 10 and 11 of glycoside hydrolases (GH) and often display low tolerance to substitutions [7]. GH10 xylanases can accommodate substitutions at + 1 subsite, but not at − 2, − 1, and + 2 subsites [8], whereas the active site of GH11 enzymes requires at least three non-substituted residues *in tandem* for catalysis [9]. Thus, GH10 and GH11 xylanases demand the prior removal of Ara*f* decorations by α-l-arabinofuranosidases to best convert xylan into xylooligosaccharides.

α-l-Arabinofuranosidases (Abfs) hydrolyze non-reducing Ara*f* groups of polysaccharides such as arabinoxylans and arabinans. They are mainly found in the GH families 43, 51, 54 and 62 and proven to have a positive effect on the enzymatic hydrolysis of pretreated wheat straw [7, 10]. In general, the characterized Abfs from family 62 seem to be specialized in cleaving Ara*f* residues from mono-substituted xylopyranosyl (Xyl*p*) units [11], whereas, in family GH43, some enzymes are specific for mono-substitutions [12, 13] and others recognize O3-linked Ara*f* moieties from di-substituted xylan [14–16]. More generalist Abfs that release Ara*f* from both mono- and di-substituted Xyl*p* residues have been found in GH51 and GH54 families [17–20]. However, the molecular adaptations that allowed some GH51 Abfs to cleave di-substitutions remain elusive. In addition, the capacity of these enzymes to cleave di-substitutions has been mainly analyzed qualitatively without a kinetic characterization using di-substituted arabinoxylooligosaccharides (AXOs) [13, 19–22].

Thus, in this work, we reveal a novel generalist GH51 enzyme that forms trimers in solution and can cope with both mono- and di-substitutions in arabinoxylans, with biotechnological potential for biomass saccharification. For the first time, the kinetic characterization by mass spectrometry was described for a di-substituted AXO and the structural basis for di-substitution recognition in the GH51 family was elucidated.

## Results

### *Xac*Abf51 is a thermotolerant α-l-arabinofuranosidase and enhances sugarcane bagasse saccharification

**Fig. 1:**
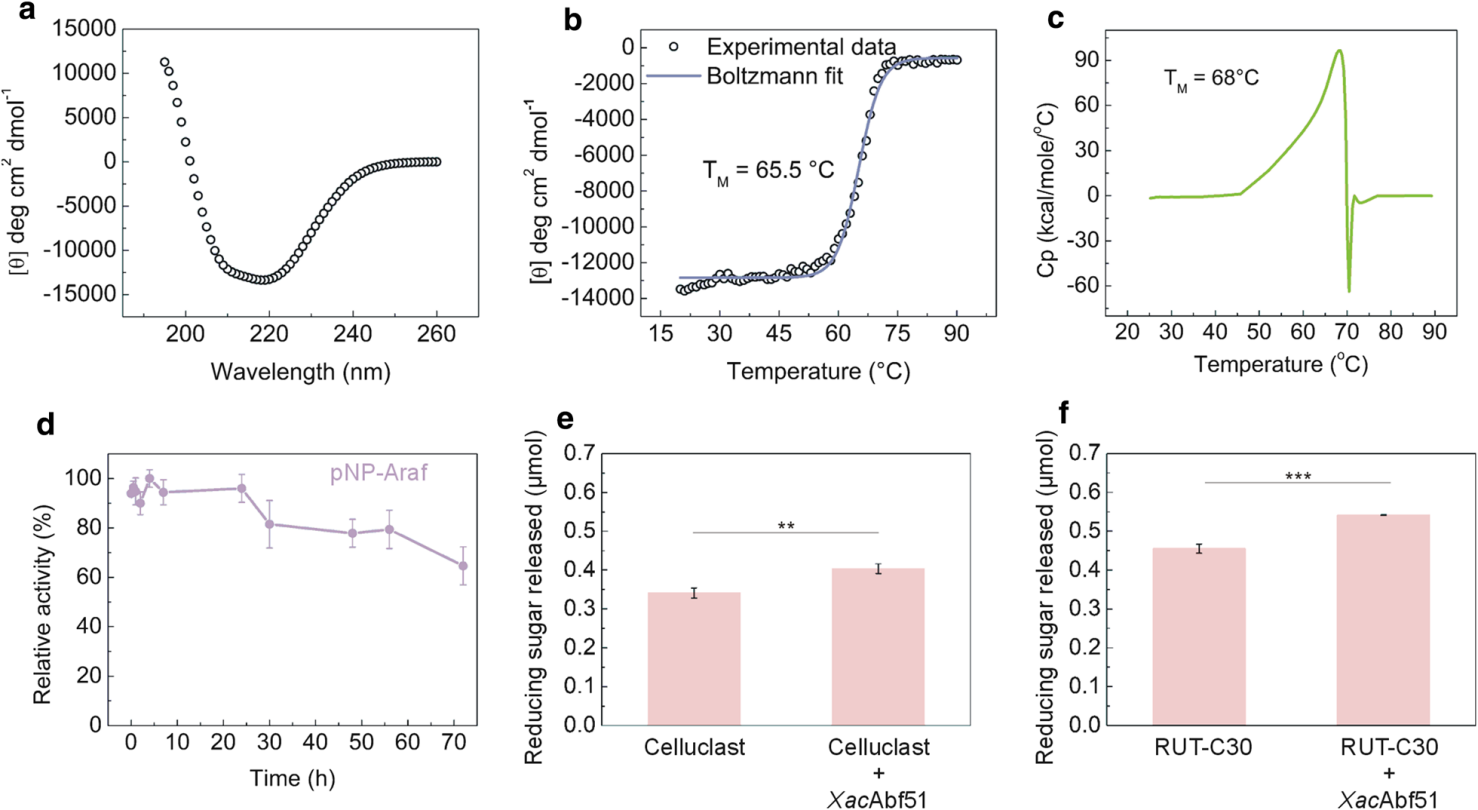
*Xac*Abf51 is a thermotolerant Abf and enhances saccharification of delignified sugarcane bagasse. Circular dichroism spectrum of *Xac*Abf51 (**a**) and thermal denaturation profile of the enzyme assessed by CD (**b**) and DSC (**c**). Residual activity of *Xac*Abf51 over pNP-Ara*f* after incubation at 50 °C for up to 72 h (**d**). Sugar released from delignified sugarcane bagasse by Celluclast (238 µg) (**e**) or *T. reesei* RUT-C30 enzyme cocktail (238 µg) (**f**) in the absence or presence of *Xac*Abf51 (13 µg). **P value ≤ 0.01; ***P value ≤ 0.001 (one-tailed Student’s *t* test)

The enzyme *Xac*Abf51 fused to an N-terminal His-tag was recombinantly expressed in *Escherichia coli* cells and purified to homogeneity by metal-affinity and size-exclusion chromatography. The melting temperature (*T*_m_) assessed by circular dichroism spectroscopy (CD) and differential scanning calorimetry (DSC) is around 67 °C (Fig. 1a–c), indicating enhanced thermotolerance compared to other glycoside hydrolases from *X. axonopodis* pv. *citri*, which usually have a *T*_m_ between 45 and 55 °C [23]. *Xac*Abf51 cleaves the synthetic substrate pNP-Ara*f*, which confirms its α-l-arabinofuranosidase activity (EC 3.2.1.55). It is very stable over time, remaining active up to 45 days, when stored at 4 °C (not shown), and retaining more than 80% of its activity after 55 h incubated at 50 °C (Fig. 1d).

The prominent thermotolerance and activity of *Xac*Abf51 in conditions akin to those used for enzymatic hydrolysis in biorefineries led us to evaluate the biotechnological potential of this novel Abf as a complement in fungal enzyme cocktails used for sugarcane bagasse degradation, since arabinoxylan is an important component of this biomass [3]. As expected, the addition of *Xac*Abf51 in celluclast and RUT-C30 enzyme cocktails enhanced the hydrolysis of delignified sugarcane bagasse in near 20%, indicating that *Xac*Abf51 might be a useful additive in enzyme formulations for sugarcane bagasse saccharification (Fig. 1e and f).

### *Xac*Abf51 recognizes internal di-substituted Xyl*p* residues

**Fig. 2:**
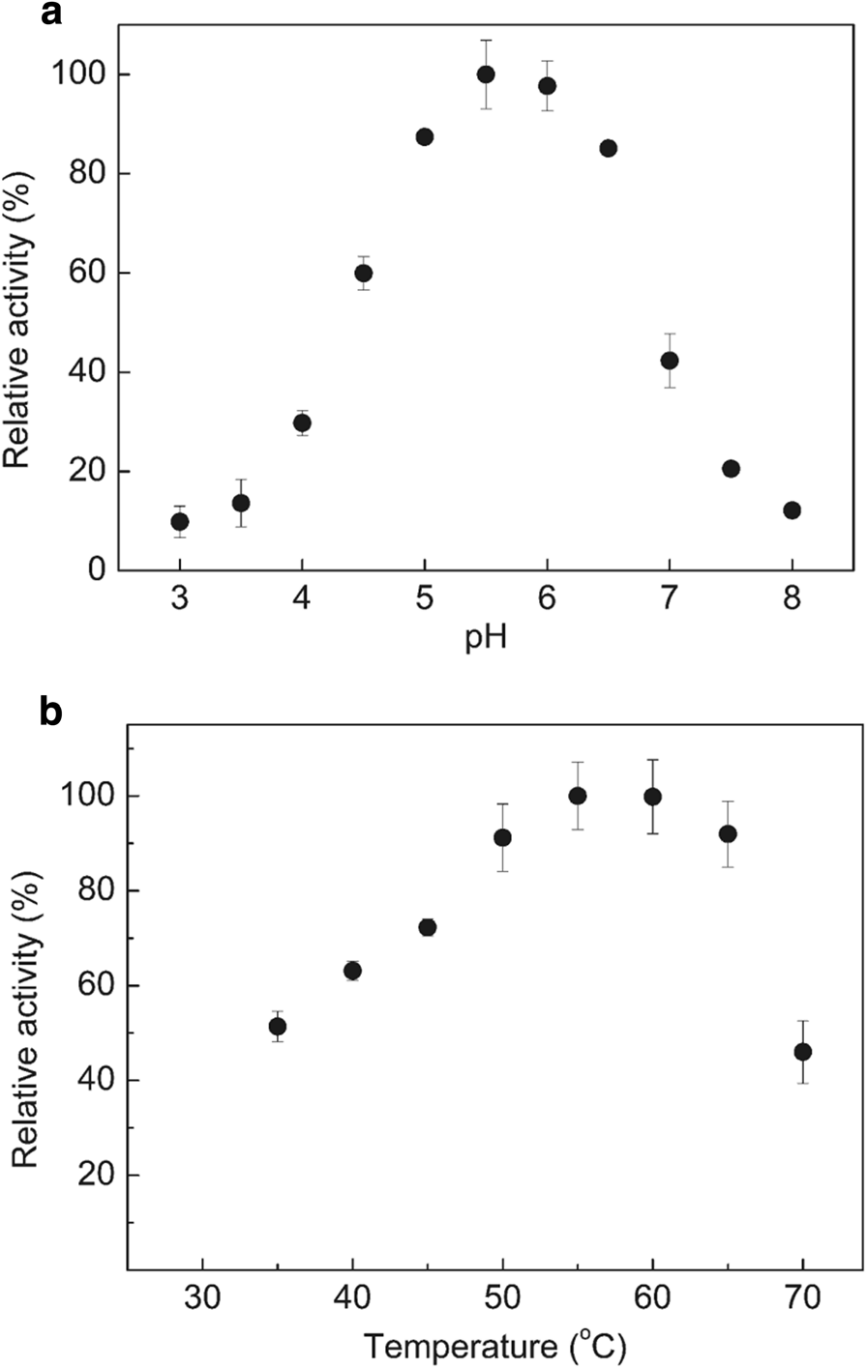
*Xac*Abf51 displays maximum activity at pH 5.5 and between 55 and 60 °C. Relative activity of *Xac*Abf51 over pNP-Ara*f* in function of pH (**a**) and temperature (**b**). Note that the optimal ranges of pH and temperature for *Xac*Abf51 activity are compatible with commercial fungal enzyme cocktails for lignocellulose saccharification

To better understand the catalytic properties of *Xac*Abf51, we characterized the influence of pH and temperature on enzyme activity and investigated its substrate specificity. Maximum catalytic rates were observed at pH 5.5 (Fig. 2a) and temperature between 55 and 60 °C (Fig. 2b), which is fully compatible with the reaction conditions of commercial fungal enzyme cocktails. Besides pNP-Ara*f*, *Xac*Abf51 also cleaves natural polysaccharides such as arabinoxylan and arabinan (Table 1). A comparison of the reaction with arabinan and arabinoxylan at 10 mg mL^−1^ indicates that the enzyme cleaves arabinan better than arabinoxylan. The enzyme was not able to cleave pNP-Xyl*p* and arabinogalactan, indicating a high specificity for Ara*f* residues linked to xylan or arabinan backbones.

**Table 1:** Kinetic parameters of *Xac*Abf51 and *Tx*AbfD3 on pNP-Ara*f* and arabinan and comparative activity of *Xac*Abf51 and *Tx*AbfD3 on arabinoxylan.

	pNP-Ara*f*			Arabinan			Arabinoxylan^a^
	*K*_m_ (mM)	*k*_cat (_s^−1^)	*k*_cat_/*K*_m_ (s^−1^ M^−1^)	*K*_m_ (mg mL^−1^)	*k*_cat_ (s^−1^)	*k*_cat_/*K*_m_ (s^−1^ mg^−1^ mL)	*v*_0_/[*E*]_*t*_ (s^−1^)
*Xac*Abf51	0.19 ± 0.01	56.4 ± 0.8	3 × 10^5^	20.4 ± 7.8	56.7 ± 6.4	2.8	9.1 ± 0.55
*Tx*AbfD3	0.52 ± 0.06	154.2 ± 4.2	3 × 10^5^	41 ± 9.5	27.15 ± 2.2	0.7	30.0 ± 1.31

For comparative purposes, the assays were performed at 50 °C, pH 5.5, which is compatible with industrial processes of biomass saccharification. The *K*_m_ and *k*_cat_ could not be estimated for arabinoxylan because the maximum velocity is not reached at the highest possible concentration of the substrate

^a^Activity measured using substrate at 10 mg mL^−1^

The higher activity of *Xac*Abf51 on arabinan as compared to arabinoxylan prompted us to investigate whether the enzyme *Tx*AbfD3 (EC 3.2.1.55) from *T. xylanilyticus*—a GH51 member highly active on arabinoxylan [24]—displays the same behavior. In contrast to *Xac*Abf51, the enzyme *Tx*AbfD3 was more active on arabinoxylan than on arabinan, showing that distinct substrate preferences occur within the family GH51, despite their capacity to recognize several substrates.

**Fig. 3:**
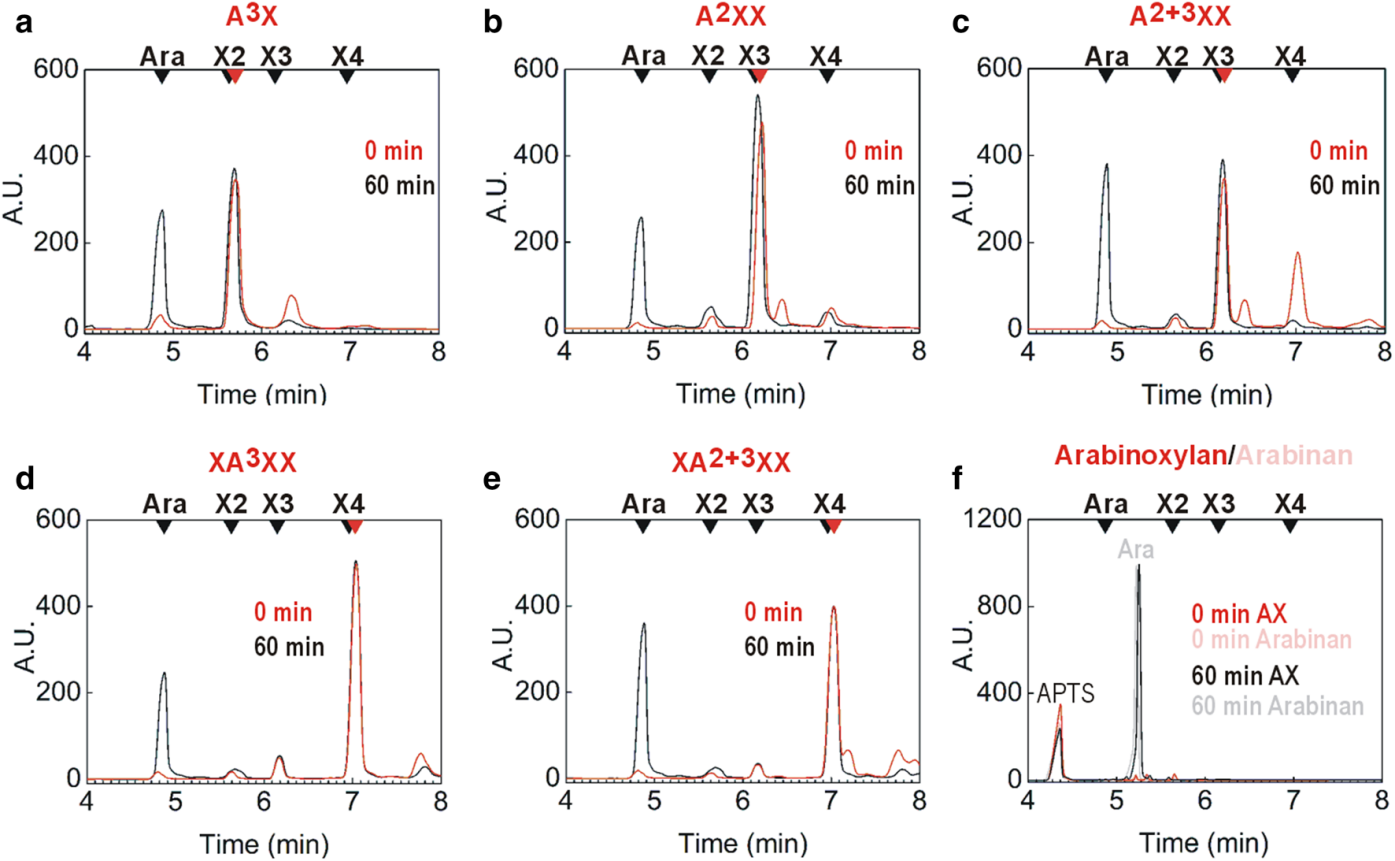
*Xac*Abf51 releases Ara*f* from mono- and di-substituted AXOS and from arabinoxylan. Capillary zone electrophoresis profiles of AXOS before (red lines) and after (black lines) incubation with *Xac*Abf51. Although the peaks of decorated and undecorated oligosaccharides were indistinguishable in this assay, the increase of arabinose (Ara) peak after enzyme treatment shows the capacity of *Xac*Abf51 to release Ara*f* from several AXOS and from arabinoxylan and arabinan. **a** A^3^X = 3^2^-α-l-arabinofuranosyl-xylobiose; **b** A^2^XX = 2^3^-α-l-arabinofuranosyl-xylotriose; **c** A^2+3^XX = 2^3^, 3^3^-di-α-l-arabinofuranosyl-xylotriose; **d** XA^3^XX = 3^3^-α-l-arabinofuranosyl-xylotetraose; **e** XA^2+3^XX = 2^3^, 3^3^-di-α-l-arabinofuranosyl-xylotetraose; **f** arabinoxylan from wheat flour and arabinan from sugar beet. Black arrowheads represent the migration time of arabinose (Ara), xylobiose (X2), xylotriose (X3) and xylotetraose (X4) standard runs. Red arrowheads represent the substrate migration time (0 min, without enzyme). In (**f**), the Ara released from arabinan was used as a reference for the analysis of arabinoxylan cleavage, due to the anomalous migration of Ara in these conditions, compared to the standard run

**Fig. 4:**
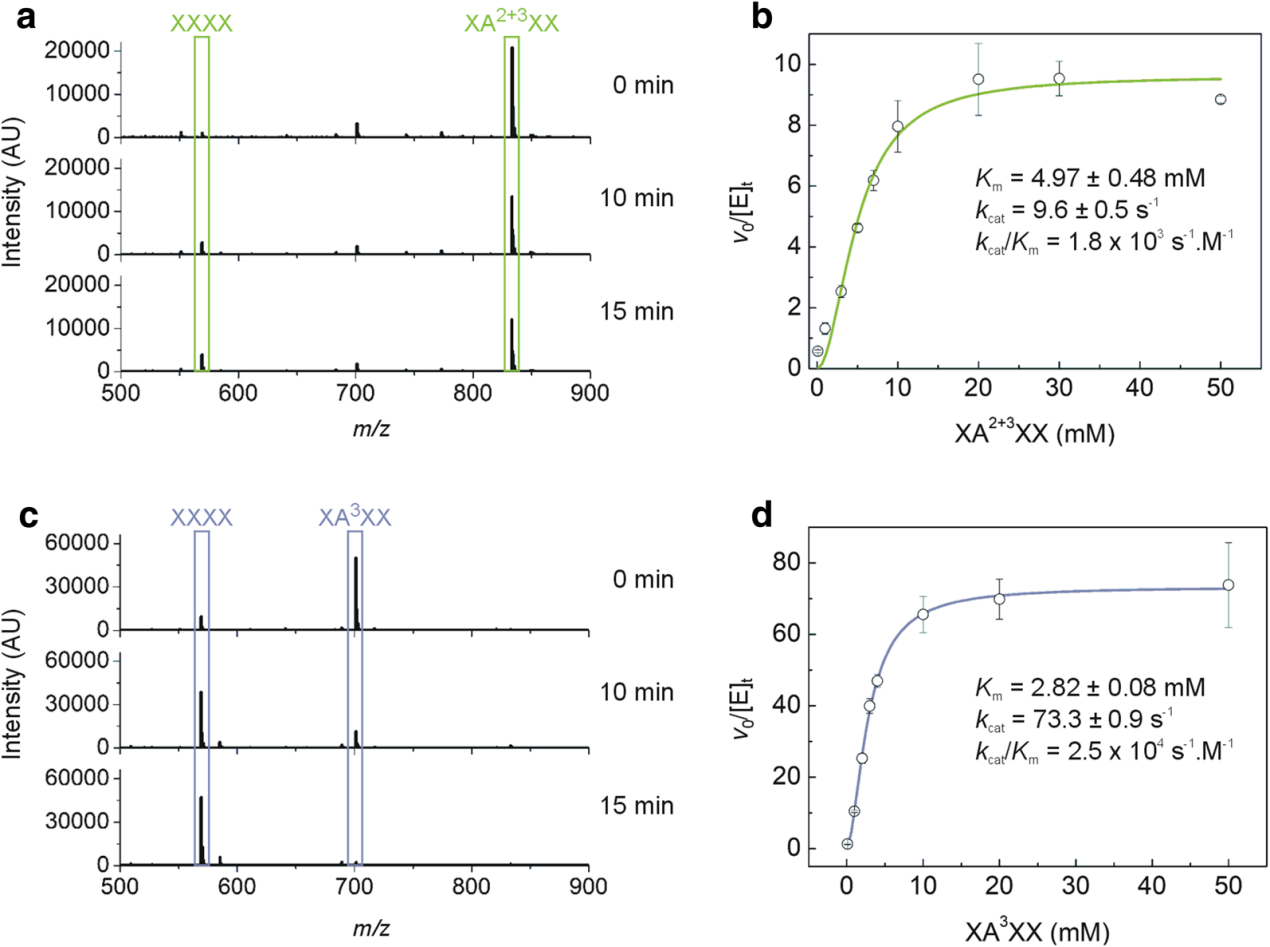
*Xac*Abf51 hydrolyzes internal mono- and di-substitutions of AXOS. **a** Mass spectra of XA^2+3^XX (2^3^, 3^3^-di-α-l-arabinofuranosyl-xylotetraose) after 0, 10 and 15 min of reaction with *Xac*Abf51 (15 µg mL^−1^) at 50 °C, pH 5.5, using 10 mM substrate. Boxed peaks correspond to the sodiated forms of the substrate XA^2+3^XX (m z^−1^ = 810 + 23 (Na^+^) = 833) and the product xylotetraose (XXXX, *m/z* = 546 + 23 (Na^+^) = 569). **b** Kinetic parameters of XA^2+3^XX hydrolysis by *Xac*Abf51 (15 µg mL^−1^) at 50 °C, pH 5.5, assessed by mass spectrometry in triplicate. **c** Mass spectra of XA^3^XX (3^3^-α-l-arabinofuranosyl-xylotetraose) after 0, 10 and 15 min of reaction with *Xac*Abf51 in the same conditions described in (**a**). Boxed peaks correspond to the sodiated forms of the substrate XA^3^XX (m z^−1^ = 678 + 23 (Na^+^) = 701) and the product XXXX (*m/z* = 546 + 23 (Na^+^) = 569). **d** Kinetic parameters of XA^3^XX hydrolysis by *Xac*Abf51 (15 µg mL^−1^) at 50 °C, pH 5.5, assessed by mass spectrometry in triplicate. Error bar represents standard deviations of the mean

According to capillary zone electrophoresis data, *Xac*Abf51 releases arabinose from arabinoxylan as well as from mono- and di-substituted Xyl*p* residues located at the non-reducing end or within the backbone of AXOS (Fig. 3). To better characterize the action of *Xac*Abf51 on internal (di)-substitutions, we monitored the enzymatic hydrolysis of 2^3^, 3^3^-di-α-l-arabinofuranosyl-xylotetraose (XA^2+3^XX) and 3^3^-α-l-arabinofuranosyl-xylotetraose (XA^3^XX) using mass spectrometry. In the reactions containing XA^2+3^XX as substrate, we detected the product xylotetraose but not mono-substituted AXOS, indicating that *Xac*Abf51 cleaves both Ara*f* moieties from internal di-substitutions (Fig. 4a). The enzyme showed a *k*_cat_ of 9.6 ± 0.5 s^−1^ and *K*_m_ = 4.97 ± 0.48 mM against the di-substituted substrate (*k*_cat_/*K*_m_ = 1.8 × 10^3^ s^−1^ M^−1^), although higher specificity was observed for the O3-linked mono-substitution (*k*_cat_= 73.3 ± 0.9 s^−1^, *K*_m_ = 2.82 ± 0.08 mM, *k*_cat_/*K*_m_ = 2.5 × 10^4^ s^−1^ M^−1^), which agrees with the lack of detection of mono-substituted intermediates in the XA^2+3^XX hydrolysis (Fig. 4a). The homologous enzyme *Tx*AbfD3 (15 µg mL^−1^), which was active against XA^3^XX (*v*_0_/[*E*]_*t*_ = 88.5 ± 2.6 s^−1^ at 10 mM substrate), displayed 20-fold lower activity against the di-substituted substrate XA^2+3^XX (*v*_0_/[*E*]_*t*_ = 0.40 ± 0.03 s^−1^ at 10 mM substrate) compared to *Xac*Abf51 (*v*_0_/[*E*]_*t*_ = 7.96 ± 0.85 s^−1^) assayed in the same conditions, indicating that *Xac*Abf51 underwent molecular adaptations to better cleave di-substituted AXOS.

### Structural basis for the cleavage of AX di-substitutions by *Xac*Abf51

**Fig. 5:**
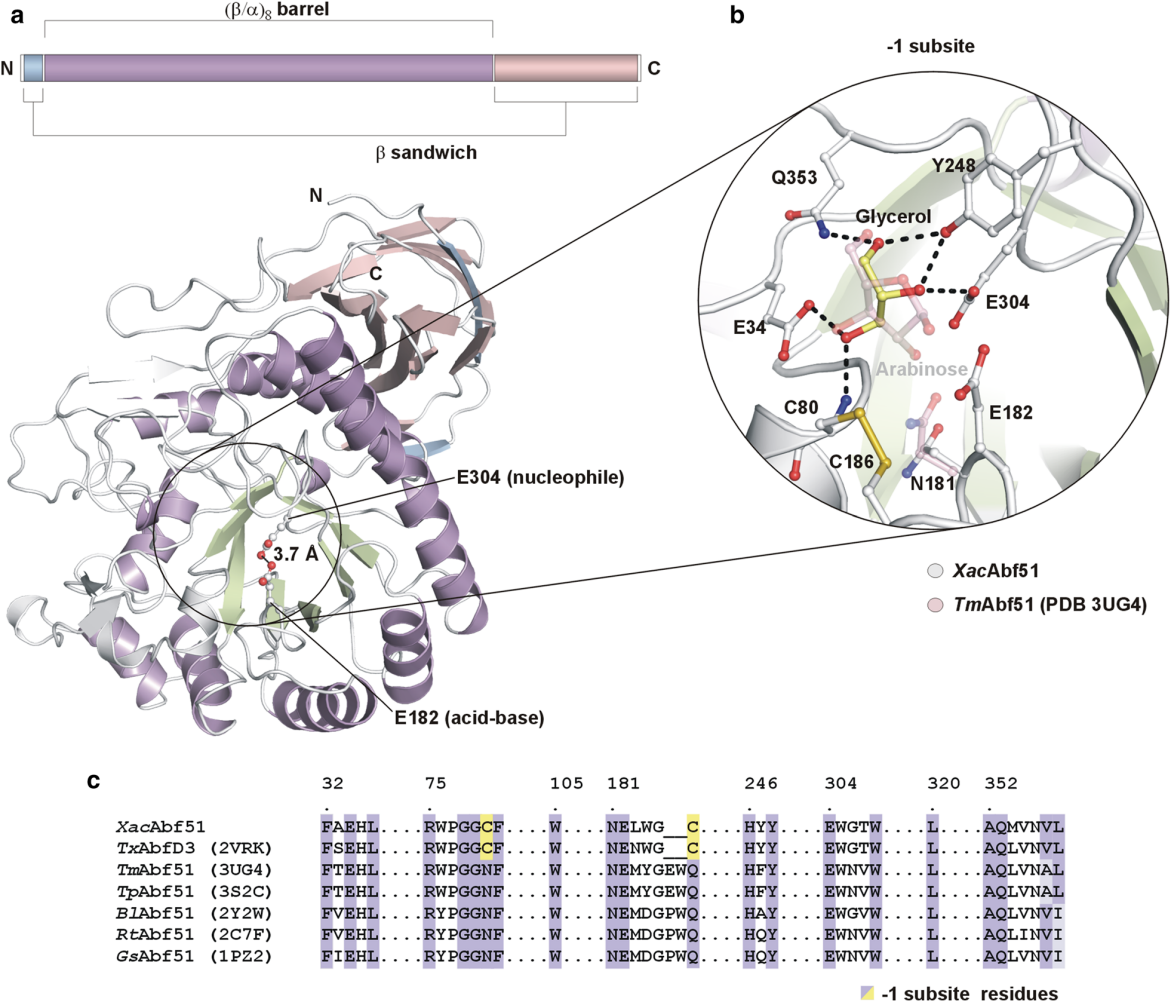
Crystallographic structure of *Xac*Abf51 reveals a typical fold of GH51 arabinofuranosidases and a disulfide bridge at − 1 subsite conserved in *Tx*AbfD3, but divergent in other structurally characterized GH51 enzymes. **a** Scheme of *Xac*Abf51 domain architecture (top) and cartoon representation of the 3D structure (bottom) highlighting the distance (3.7 Å) between the catalytic residues (sticks) compatible with the retaining mechanism of hydrolysis found in GH51 family. **b** Magnified view of − 1 subsite (ball and sticks, light gray C atoms) in which a glycerol molecule (yellow C atoms) is bound mimicking part of the arabinose scaffold observed in the crystallographic structure of *Tm*Abf51–arabinose complex (pink C atoms). **c** Structure-based sequence alignment of − 1 subsite (boxed residues) from the GH51 enzymes of known structure. Dark violet represents identical residues, light violet semi-conserved and yellow highlights the cysteine residues that form a disulfide bridge only in the *Xac*Abf51 and *Tx*AbfD3 enzymes of the presented comparison. *Tm*: *Thermotoga maritima*; *Tp*: *Thermotoga petrophila*; *Bl*: *Bifidobacterium longum*; *Rt*: *Ruminiclostridium thermocellum; Gs*: *Geobacillus stearothermophilus*

To investigate the molecular mechanisms by which *Xac*Abf51 cleaves AX di-substitutions, we solved and analyzed its crystal structure. As a typical GH51 enzyme, *Xac*Abf51 harbors the active site in a (*β*/*α*)_8_-barrel that is tightly associated with a β-sandwich domain. The β-sheets of this β-sandwich put the N- and the C-terminal regions of the barrel together, stabilizing these two regions that otherwise would be labile (Fig. 5a). Thus, although not participating in the catalysis, the β-sandwich domain seems to be essential for the catalytic domain stability.

Structural comparisons revealed that *Xac*Abf51 displays all structural features required for the retaining mechanism of hydrolysis conserved in GH51 enzymes [25–27]. The catalytic residues Glu182 (acid–base) and Glu304 (nucleophile) are positioned 3.7 Å apart from each other within the active site pocket (Fig. 5a). A glycerol molecule occupied the − 1 subsite in a conformation that mimics part of the Ara*f* ring (Fig. 5b). All residues from this subsite are identical or semi-conserved between *Xac*Abf51 and GH51 structures known so far, except for Cys80 and Cys186. These cysteine residues form a disulfide bridge in *Xac*Abf51 and *Tx*AbfD3, which likely contributes to the high thermostability of these enzymes [24]. In other GH51 Abfs, Cys80 and Cys186 residues are replaced by asparagine and glutamine (Fig. 5c). Although Asn181 is fully conserved between the compared GH51 Abfs, it adopts a different rotamer in *Xac*Abf51 (Fig. 5b).

**Fig. 6:**
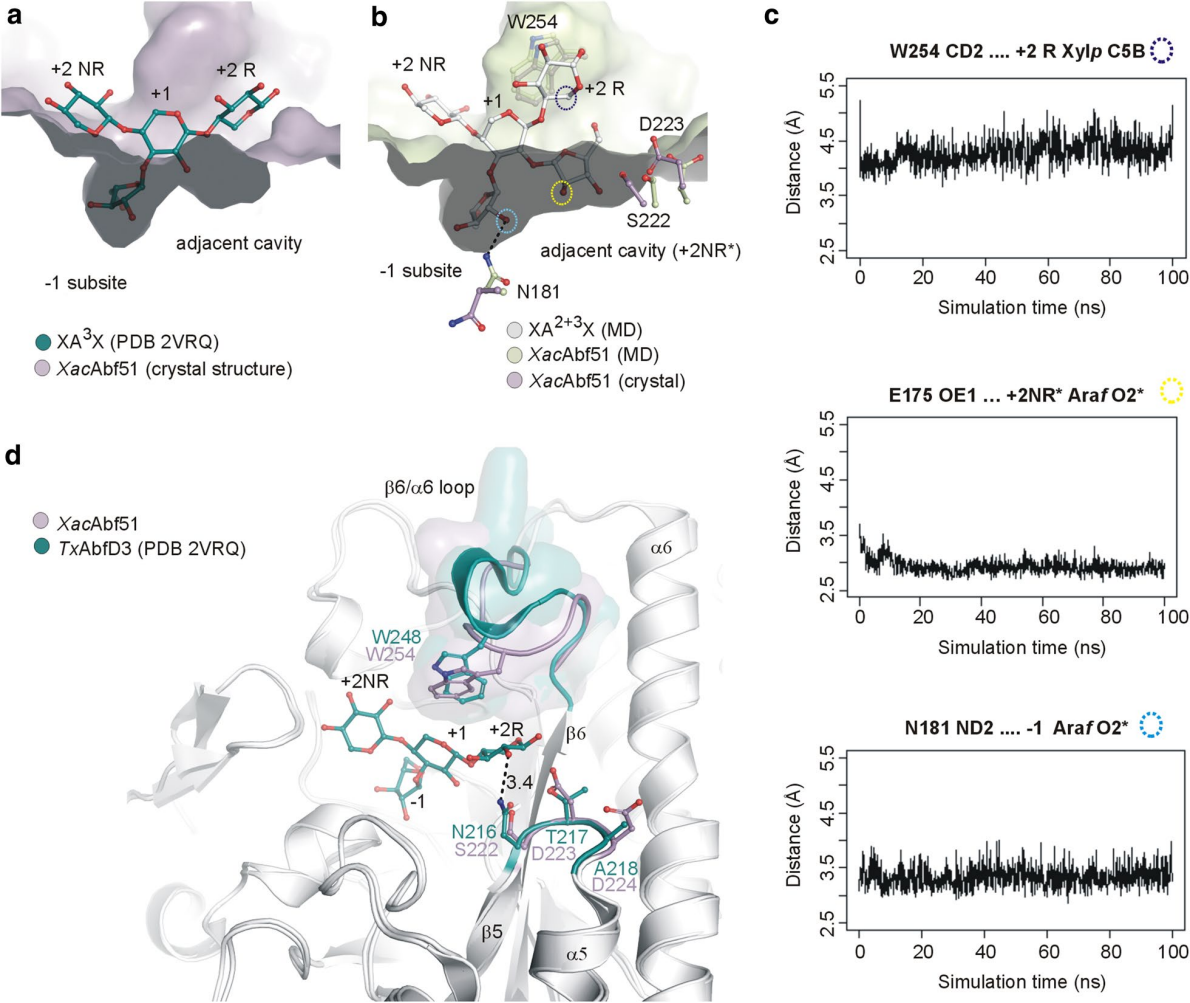
A cavity adjacent to − 1 subsite accommodates the second decoration of di-substituted AXOS. **a** Structural superposition of *Xac*Abf51 structure (violet surface) with *Tx*AbfD3 structure in complex with 3^2^-α-l-arabinofuranosyl-xylotriose (XA^3^X; blue C atoms). Subsites are labeled according to the nomenclature used by McKee and coworkers [14]. *NR* non-reducing end, *R*  reducing end. **b** Comparison between *Xac*Abf51 crystal structure and the modeled *Xac*Abf51–XA^2+3^X complex after 100 ns of molecular dynamics simulation. According to this simulation, the xylan backbone bends at the β-1,4 linkage involving the reducing end of substrate to better accommodate the di-substitution in the cavity adjacent to the − 1 subsite of *Xac*Abf51. **c** Selected inter-atomic distances between enzyme and substrate indicate favorable interactions over the simulation. Colored circles refer to the selected substrate atoms highlighted in **b** (open circles). **d** Structural comparison of *Xac*Abf51 and *Tx*AbfD3 crystal structures highlighting the divergent loops β5–α5 and β6–α6 that delineates the +1 and +2R subsites. The substrate XA^3^X bound to *Tx*AbfD3, as well as the different positioning of W254 compared to W248 and the side chains of SDD and NTA motifs are shown in sticks and color-coded according to the respective structure. Note the hydrogen bond between N216 and the O2 of +2R Xyl*p* residue that is absent in *Xac*Abf51

Structural superimposition of *Xac*Abf51 with *Tx*AbfD3 in complex with 3^2^-α-l-arabinofuranosyl-xylotriose (XA^3^X) evidenced the presence of a cavity near to the − 1 subsite that could potentially accommodate the second Ara*f* substitution of a di-substituted substrate (Fig. 6a). To gain insights into the molecular events involved in binding and hydrolysis of Ara*f* from internal di-substituted Xyl*p* residues, we appended an O2-linked Ara*f* at XA^3^X, thus generating XA^2+3^X, and carried out a molecular dynamics (MD) simulation of *Xac*Abf51 complexed with this di-substituted substrate. According to this simulation, the side chains of Ser222 and Asp223 adopted different rotameric conformations to better accommodate the O_2_-linked Ara*f* at the +2NR* subsite (Fig. 6b). The side chain of Asn181 rotated 180° around Cβ to interact with the O_2_ atom of the arabinofuranosyl residue at the − 1 subsite. Trp254 formed hydrophobic interactions with the +2R Xyl*p* residue, but no hydrogen bonds were observed between the enzyme and the xylan backbone, which correlates with the versatility of *Xac*Abf51 in recognizing both arabinoxylan and arabinan. Selected inter-atomic distances between enzyme and XA^2+3^X remained stable over the simulation, indicating favorable interactions for substrate binding (Fig. 6c). Thus, the MD simulation data support that the pocket adjacent to − 1 subsite can accommodate the O2-linked Ara*f* from internal di-substituted Xyl*p* residues, while the O3-linked decoration is placed into − 1 subsite for catalysis. Considering the pseudosymmetry of xylan and the design of catalytic interface, the backbone might also bind to the active site in the inverted direction, placing, in this case, the internal O2-linked Ara*f* (from mono- or di-substitutions) into − 1 subsite for cleavage.

In *Tx*AbfD3, we observed variable regions at β6–α6 and β5–α5 loops that might explain its low activity against internal di-substitutions. The β6–α6 loop contains the tryptophan residue that interacts with the + 1 Xyl*p* unit in *Tx*AbfD3, but makes hydrophobic contacts with the + 2 Xyl*p* residue in *Xac*Abf51 (Fig. 6d). To test the influence of β6–α6 loop in substrate preference, the sequence TIPGGWPPRASST (Thr249-Thr261) and two extra residues (Ala310-Pro311) of *Xac*Abf51 were replaced by the sequence TVPGPWEKKGPAT and DV of *Tx*AbfD3, because the aspartic residue from the DV motif interacts with β6–α6 loop in *Tx*AbfD3. CD analysis indicated a folded conformation of the mutant (data not shown); however, it was inactive against arabinan and arabinoxylan and poorly active against pNP-Ara*f*. Another point of divergence between *Xac*Abf51 and *Tx*AbfD3 is the sequence SDD (Ser222-Asp224, β5–α5 loop) of *Xac*Abf51, which is replaced by the NTA (Asn216-Ala218) motif in *Tx*AbfD3, attracting the +2 Xyl*p* unit via a hydrogen bond donated by Asn216 (Fig. 6d). This three-residue replacement caused enzyme aggregation, as assessed by Dynamic Light Scattering (DLS), and disrupted the enzyme activity against arabinoxylan and arabinan (results not shown). We also tested whether the triple replacement of β6–α6 loop, DV and SDD motifs would convert the substrate preference of *Xac*Abf51 to that of *Tx*AbfD3. Although the mutant showed a folded conformation with a similar hydrodynamic radius (*R*_h_) to the WT enzyme, the triple modification also abolished the *Xac*Abf51 activity against arabinoxylan and arabinan, indicating that other structural features might affect the positioning and dynamics of β6–α6 and β5–α5 loops, impairing activity when associated with transplanted loops.

### The biological unit of *Xac*Abf51 is a trimer

**Fig. 7:**
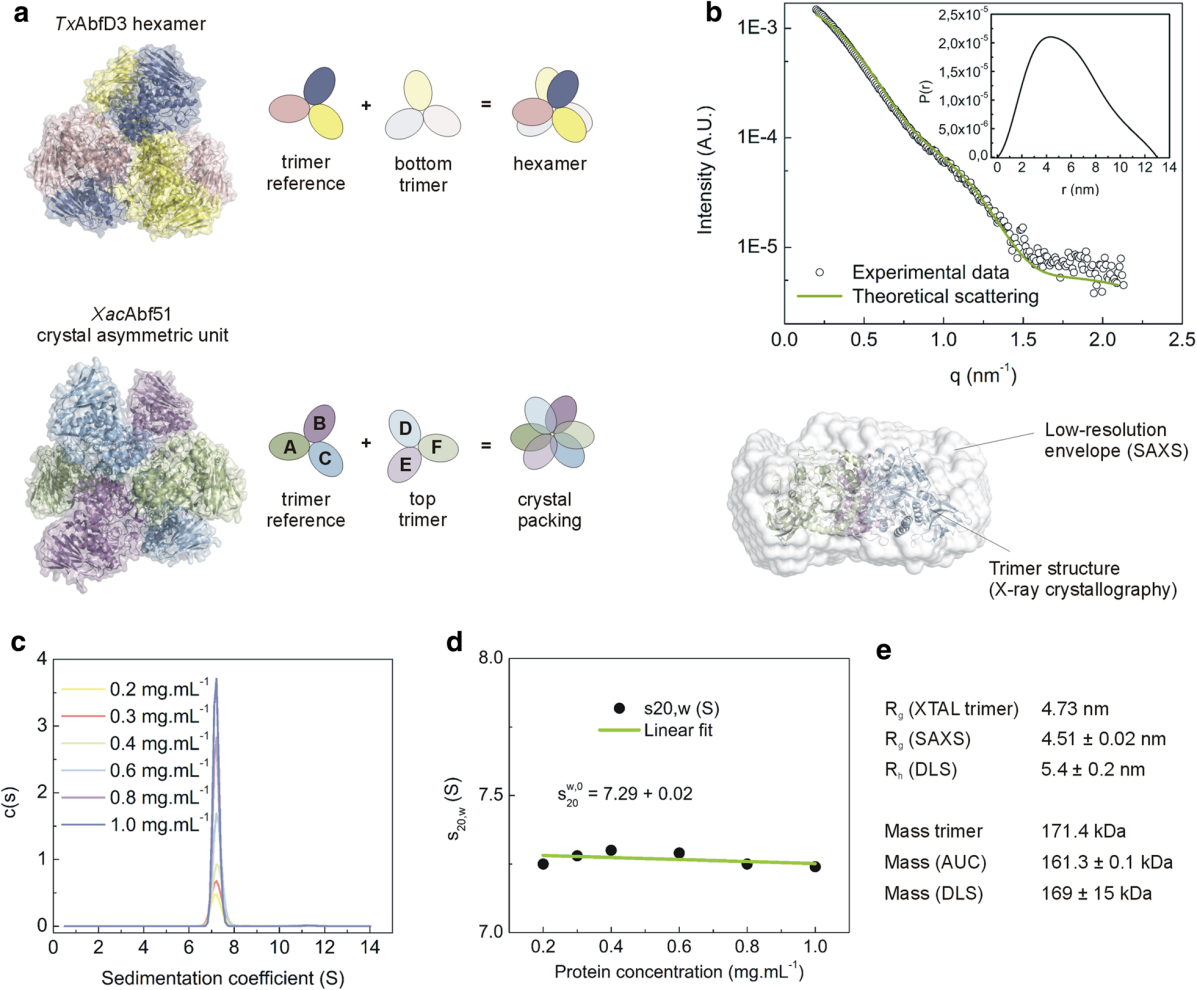
*Xac*Abf51 is a trimeric enzyme. **a** Comparison of *Tx*AbfD3 hexamer with the molecules found in the asymmetric unit of *Xac*Abf51 crystal (cartoon with transparent surface). The schemes highlight that in the *Xac*Abf51 crystal structure the ABC and DEF trimers interact with each other in a different way compared to the trimer–trimer interface of *Tx*AbfD3 hexamer. **b** SAXS curve (open circles) agrees with the theoretical profile of *Xac*Abf51 trimer calculated from the crystal structure using CRYSOL. The inset shows the pair–distance distribution function computed from the experimental data and used to generate the low-resolution envelope (white surface) fitted to the crystallographic trimer (cartoon). **c**, **d** AUC data show that *Xac*Abf51 assumes a trimer arrangement in a wide range of protein concentration. **e** Summary of size and mass parameters estimated using four biophysical techniques demonstrates that the quaternary structure of *Xac*Abf51 is a trimer

In the crystal structure of *Xac*Abf51, six protein chains compose the asymmetric unit, but in a different spatial disposition from that observed for known GH51 hexamers such as *Tx*AbfD3 [28] (Fig. 7a). Analysis of the crystal interfaces using jsPISA [29] indicates that trimers, composed by ABC or DEF chains, are the most stable quaternary structure of *Xac*Abf51. Moreover, the interface between the dimer of trimers that compose the *Tx*AbfD3 hexamer is not conserved in *Xac*Abf51.

To determine the oligomeric state of *Xac*Abf51 *in solution*, several experiments were carried out with the purified protein. The small angle X-ray scattering (SAXS) curve of *Xac*Abf51 revealed a radius of gyration (4.5 nm) and a low-resolution molecular envelope that are consistent with the crystallographic trimer (Fig. 7b). Moreover, the sedimentation coefficient estimated from analytical ultracentrifugation (AUC) at different protein concentrations (Fig. 7c and d) corresponds to a particle of 161 kDa, which is in accordance with the theoretical mass of the trimer (171 kDa). Estimation of *R*_h_ using DLS (Fig. 7e) further supported that the biological unit of *Xac*Abf51 is a trimer.

### Evolution of GH51 enzymes

**Fig. 8:**
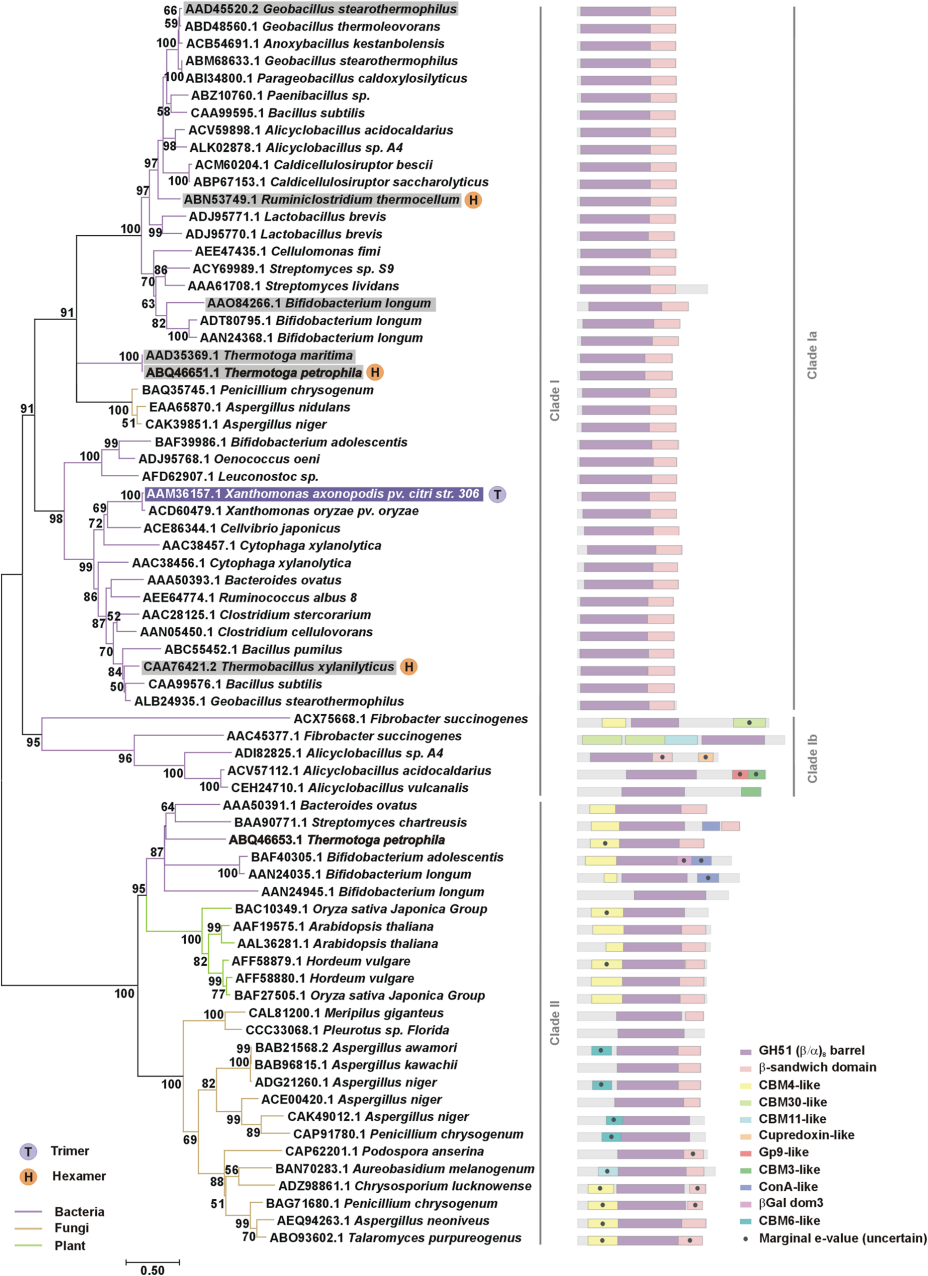
Molecular phylogenetic analysis of GH51 family. Phylogenetic tree (unrooted) based on a multiple sequence alignment of the (*β*/*α*)_8_ barrel of characterized GH51 enzymes present in the CAZY database [7] and the respective paralogues. The evolutionary history was inferred using the maximum likelihood method implemented in the MEGA7 software [65, 67]. The tree with the highest log likelihood (− 23,649.75) is shown and the percentage of trees in which the associated taxa clustered together are shown next to the branches (except for those with values below 50%). Branch lengths represent the number of substitutions per site. The right panel shows the domain architecture predicted for each sequence using the webserver SUPERFAMILY [63]. Proteins with known 3D structure are highlighted with purple (*Xac*Abf51) or gray boxes. Paralogous sequences from *T. petrophila* are shown in bold

To gather insight into the evolution of GH51 Abfs, a phylogenetic tree was constructed based on the catalytic domain of characterized GH51 enzymes and their respective paralogues (Fig. 8). This phylogenetic reconstruction shows two major clades (clades I and II) referent to a gene duplication that occurred early in evolution, as indicated by the presence of genes from the two clades in *Thermotoga petrophila*, a species from a deep phylogenetic branch in the tree of life [30]. Members of clade I are abundant in bacteria, whereas those of clade II are found mainly in plants and fungi.

The division in two major clades reflects two main types of modular architecture. In clade I, most enzymes display the (*β*/*α*)_8_ barrel + β-sandwich composition, but, in clade II, the proteins have an extra N-terminal domain which resembles carbohydrate-binding modules (CBM) from families 4, 6 or 11 (Fig. 8). Interestingly, enzymes with β-1,4-glucanase activity, found only in specific bacteria from *Fibrobacter* and *Alicyclobacillus* genera (clade Ib), have peculiar and diverse domain arrangements, indicating they emerged from gene duplication and recombination events. In these enzymes, the (*β*/*α*)_8_ barrel is usually fused to one or more copies of putative cellulose-binding modules (CBM 3, 11 and 30). Moreover, unconventional domains (Gp9-like and cupredoxin-like) are detected in two endoglucanases from *Alicyclobacillus* sp.

To date, the only structures available for the GH51 family comprise Abfs from clade Ia with the (*β*/*α*)_8_ barrel + β-sandwich composition. Except for *Xac*Abf51, which is a trimer, the other structures reported so far are hexamers, indicating that the molecular diversity of GH51 enzymes include changes in quaternary structure besides modular rearrangements. The capacity to cleave α-1,2 and α-1,3 Ara*f* decorations in arabinoxylan and/or arabinan as well as α-1,5 bonds in arabinan is observed in Abfs from both clades I and II, evidencing the structural plasticity of the GH51 active site [13, 20, 31–37].

## Discussion

This study reports the first Michaelis–Menten kinetic parameters for the cleavage of internal Ara*f* di-substitutions by a GH51 Abf and provides the structural basis for this activity. Cleavage of terminal di-substitutions in AXOS has been reported for some GH51 enzymes, but internal di-substitutions have been described as poor or non-cleavable substrates [17–20]. Our data reveal a novel GH51 enzyme that releases both Ara*f* residues from internal di-substitutions with a catalytic constant of ~ 10 s^−1^. Although our data do not resolve the *Xac*Abf51 preference between O2 or O3 linkages, they reveal that the first cleavage of a di-substitution is the rate-limiting step of the reaction catalyzed by *Xac*Abf51, leading to a tenfold lower *k*_cat_/*K*_m_ for the di-substituted compared to the O3-mono-substituted substrate.

For almost all GH51 enzymes characterized so far, kinetic parameters have only been assessed using synthetic substrates (pNP derivatives), probably because of the high-cost and limited availability of AXOS allied to the low response stability and time-consuming characteristic of HPAEC-PAD analyses [38]. To overcome such bottlenecks, we used mass spectrometry to monitor the enzymatic hydrolysis of mono(di)-substituted arabinoxylotetraoses—a fast, direct and highly sensitive approach that requires minimum amounts of substrate (in this study, we acquired each data point in 1 min and used less than 10 mg of substrate for a complete enzyme characterization). Thus, we envisage the mass spectrometry as a useful, fast and precise alternative, not only for future studies of GH51 enzymes, but also to assess Michaelis–Menten kinetics of oligosaccharide hydrolysis by other GHs, as previously reported for xylanases [39].

The positive effect of *Xac*Abf51 in the saccharification of delignified sugarcane bagasse may be useful for the development of enzyme cocktails optimized for this biomass. Supplementation of fungal cellulases mixtures with hemicellulases and auxiliary enzymes, including a GH51 Abf, has already been shown to increase the conversion of AFEX pretreated corn stover into monosaccharides [40]. Here we evidence that this approach is also valuable to increase the hydrolysis yield of pretreated sugarcane bagasse. The cellulolytic fungi *T. reesei* displays three Abfs (GH43, GH62 and GH54), but is devoid of GH51 enzymes [41]. Thus, our data support that the *Xac*Abf51 capacity of releasing terminal and internal di-substitutions of AXOS might improve the performance of widely used cellulolytic enzyme cocktails over arabinoxylan-rich biomasses.

**Fig. 9:**
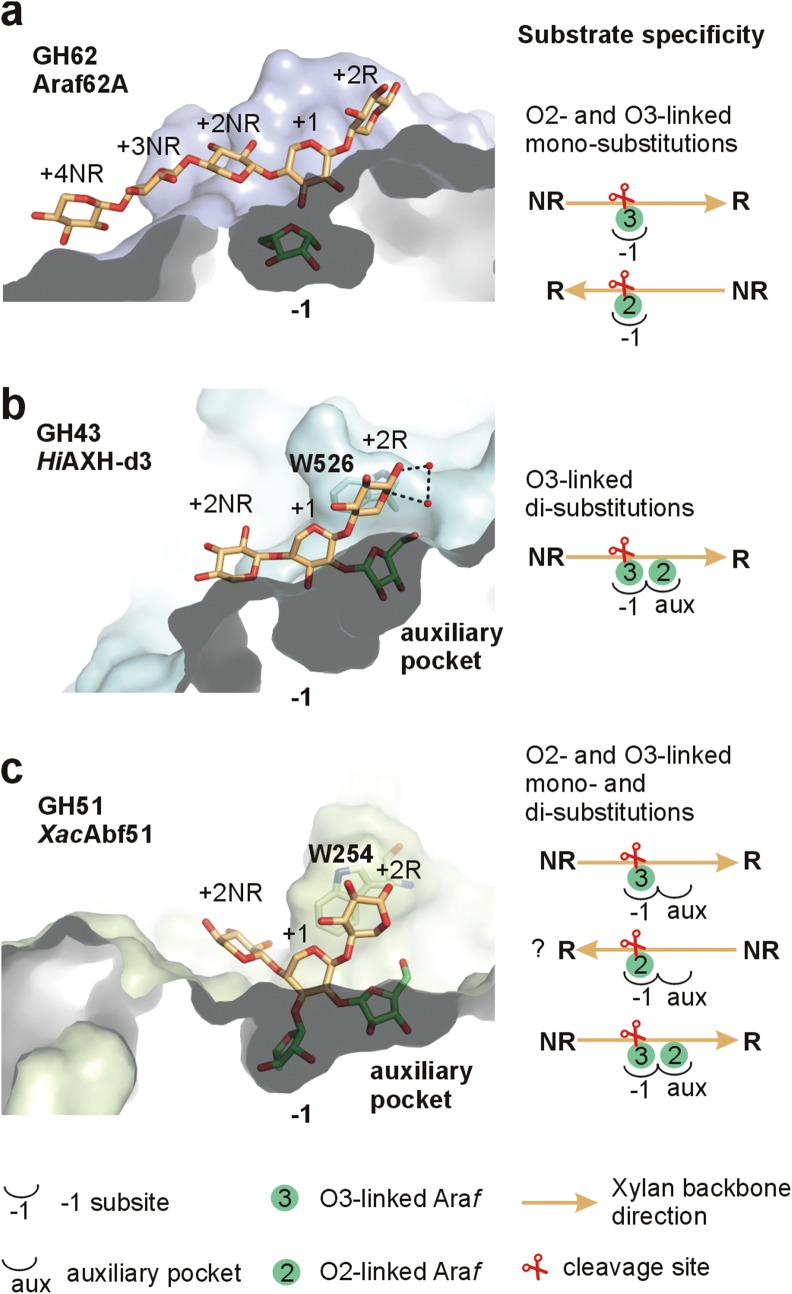
Molecular diversity of arabinoxylan-degrading mechanisms by Abfs. **a** The active site of Araf62A (GH62) is composed by a cleft that accommodates the xylan backbone and a − 1 subsite that binds specifically to mono-substitutions of Ara*f* (O2- or O3-linked). The arabinose (green C atoms) and protein surface are from PDB 3WN0, while the xylan backbone (orange C atoms) is from PDB 3WN2 [42]. *NR* non-reducing end, *R* reducing end. **b** *Hi*AXH-d3 from *Humicola insolens* (GH43) cleaves specifically the O3 linked Ara*f* substitution from di-substituted Xyl*p* units and displays an auxiliary pocket to accommodate the di-substitution. The residues W526 selects a single orientation for arabinoxylan binding into the active site via solvent-mediated interactions (dashed lines) with the endocyclic oxygen of +2R Xyl*p* (PDB 3ZXK, [14]). **c** The active site of *Xac*Abf51 also has an auxiliary pocket to accommodate di-substitutions, but the positioning of W254 seems to accept the binding of arabinoxylan in the direct and reverse direction to allow the cleavage of O3 and O2 substitutions, respectively, making this enzyme a generalist Abf

Our structural data compared to those of GH62 and GH43 Abfs (EC 3.2.1.55) contribute to a better understanding of the molecular determinants for distinct substrate specificities in Abfs. GH62 enzymes specialized in mono-substitutions display a single arabinose-binding pocket in the middle of a long cleft where the xylan backbone binds (Fig. 9a). As proposed by Maehara and coworkers, the pseudosymmetry of xylan backbone and the active site topology of Araf62A likely allows arabinoxylan to bind into the cleft in two opposite directions to, respectively, allocate the O3- and O2-linked mono-substitutions at the − 1 subsite [42]. Differently, in the GH43 enzyme *Hi*AXH-d3, which is specific for O3-linked Ara*f* from di-substitutions, an auxiliary pocket accommodates the second Ara*f* decoration and solvent-mediated hydrogen bonds (involving Trp526 and the ring oxygen of +2R Xyl*p*) selects a single orientation of the xylan backbone, in a manner that the catalytic pocket is always occupied by the O3-Ara*f* moiety (Fig. 9b) [14]. Similar to *Hi*AXH-d3, *Xac*Abf51 also displays an auxiliary pocket to accommodate the second substitution of di-substituted substrates (Fig. 9c). However, the residue Trp254 (equivalent to Trp526 of *Hi*AXH-d3) makes a π-stacking interaction with +2R Xyl*p*, which does not depend on the endocyclic oxygen, the only asymmetric feature of xylan. Thus, according to these analyses, it is plausible to suggest that the active site of *Xac*Abf51 allows the bidirectional binding of arabinoxylan and AXOS to cleave O2- and O3-linked Ara*f* from mono- or di-substitutions.

The positioning of Trp254 seems to play a role in di-substitution recognition. However, our mutational strategy to test this hypothesis (β6–α6 and/or β5–α5 loops transplantation from *Tx*AbfD3 to *Xac*Abf51) inactivated *Xac*Abf51 instead of changing its substrate specificity, indicating an incompatibility that may require secondary mutations or the reverse transplant (from *Xac*Abf51 to *Tx*AbfD3) to attain the expected functional changes.

All GH51 proteins whose structure is currently available are bacterial enzymes from Clade Ia (Fig. 8). The oligomeric state of only three of them has been validated *in solution* [*Tp*Abf51, [43]; *Tx*AbfD3 (AUC data not shown) and *Xac*Abf51 (Fig. 7)] and served as a guide to map how the quaternary structure of GH51 enzymes evolved. The hexameric arrangement, which can be seen as dimer of trimers, seems to have appeared early during evolution of GH51 family, being found in the *Thermotoga* genus, a deep lineage back to the early forms of bacteria [30, 43]. The hexameric arrangement remained stable in other thermophilic bacteria, such as *Ruminiclostridium thermocellum* (jsPISA prediction, [29]) and *T. xylanilyticus* [28]), but, in the mesophilic *X. axonopodis* pv. *citri*, the dimer of trimers was disrupted, giving rise to a trimeric enzyme. Based on these data, we suggest that the ancient GH51 arabinofuranosidases from clade I formed hexamers—possibly to withstand extreme conditions of high temperature—and that colder environments favored the emergence of trimeric enzymes, at least during *X. axonopodis* pv. *citri* speciation, changing the paradigm that GH51 Abfs are exclusively hexameric. According to ConSurf analyses [44], the trimer interface, which is close to the active site, harbors residues more conserved than those assembling trimers into hexamers, indicating that the trimeric arrangement may be more crucial than the hexameric configuration for enzyme function.

## Conclusions

In summary, our study expands our knowledge about the diversity of GH51 Abfs in terms of tertiary and quaternary structure and provides the structural basis for the release of internal Ara*f* di-substitutions by a generalist Abf that copes with all types of Ara*f* decorations in arabinoxylan and arabinan. The rare mode of action of *Xac*Abf51, along with full pH and temperature compatibility with current fungal enzyme cocktails, is very attractive for industrial applications, especially in technologies for the production of fermentable sugars using arabinoxylan-rich biomasses such as sugarcane, corn stover and grasses.

## Methods

### Molecular cloning

The nucleotide sequence encoding *Xac*Abf51 (GenBank AAM36157.1) was amplified from the genomic DNA of *X. axonopodis* pv. citri str. 306 using the following oligonucleotides: 5′- CAT ATG CCG GCC AGC TTC GCT G -3′ and 5′- AAG CTT TCA TTG CAG CTT GAG CAT CAC GAT CG -3′. It was cloned into pET28a after digestion with *Nde*I and *Hind*III restriction enzymes. The annotated sequence begins with GTG codon but upstream sequence analysis indicated additional 27 nucleotides (beginning at ATG) that are part of the signal peptide according to PROSECTO (http://www.lge.ibi.unicamp.br/lnbio/prosecto.htm). The signal peptide was removed during cloning for expression in *E. coli*. The DNA sequences of mutants I (containing 249-TVPGPWEKKGPAT-261 and 310-DV-311 instead of 249-TIPGGWPPRASST-261 and 310-AP-311), II (containing 222-NTA-224 instead of 222-SDD-224) and III (mutations I + II) were produced in pET28a vector, between *Nde*I and *Xho*I restrictions sites, by GenScript (Piscataway, NJ). The construct *Tx*AbfD3 (GenBank CAA76421.2) cloned into pET21a between *Nde*I and *Hin*dIII restriction sites was also purchased from GenScript (Piscataway, NJ).

### Protein production and purification

*Xac*Abf51 and mutants were expressed in *Escherichia coli* Origami™2(DE3) cells in Terrific broth medium (1.2% (m v^−1^) tryptone, 2.4% (m v^−1^) yeast extract, 0.4% (v v^−1^) glycerol, 17 mM sodium phosphate monobasic monohydrate, 72 mM sodium phosphate dibasic) supplemented with 50 µg mL^−1^ kanamycin. The culture was grown at 37 °C, 225 rpm, until the O.D._600nm_ has reached 1.0, transferred to 20 °C for 1 h, and then incubated with 0.25 mM isopropyl β-d-1-thiogalactopyranoside (Sigma-Aldrich, St. Louis, MO) for 18 h at 20 °C, 170 rpm. The *Tx*AbfD3 protein was produced in *E. coli* BL21 (DE3) cells grown in Luria–Bertani medium supplemented with 100 µg mL^−1^ ampicillin, following the same protocol used for *Xac*Abf51. The cells were collected, resuspended in lysis buffer (20 mM sodium phosphate, pH 7.5, 500 mM NaCl, 5 mM imidazole, 1 mM PMSF, 0.5 mg mL^−1^ lysozyme), incubated on ice for 30 min and disrupted by sonication. The soluble extract was applied into a 5-mL HiTrap Chelating HP column (GE Healthcare, Little Chalfont, UK), previously charged with Ni^2+^, coupled to an ÄKTA purifier (GE Healthcare, Little Chalfont, UK), at a flow rate of 2 mL min^−1^. The target proteins were eluted using a non-linear (0–0.5 M) gradient of imidazole. The fractions containing pure proteins were pooled, concentrated and applied into a HiLoad 16/600 Superdex 200 pg column (GE Healthcare, Little Chalfont, UK), previously equilibrated with 20 mM sodium phosphate, pH 7.5, and 150 mM NaCl, coupled to an ÄKTA purifier (GE Healthcare, Little Chalfont, UK) at a flow rate of 1 mL min^−1^.

### Enzyme activity assays monitored by colorimetric methods

Arabinose, pNP and 4-nitrophenyl-α-l-arabinofuranoside (pNP-Ara*f)* were purchased from Sigma-Aldrich, St. Louis, MO. Wheat flour arabinoxylan, sugar beet arabinan and AXOS were purchased from Megazyme, Co. Wicklow, IE.

For the thermotolerance assay, *Xac*Abf51 was incubated at 50 °C for up to 72 h and samples were collected to measure activity against pNP-Ara*f* in McIlvaine buffer (pH 5.5) at 50 °C, for 10 min, using 0.5 µg mL^−1^ (9 nM) enzyme and 10 mM substrate. The generation of *p*-nitrophenolate from pNP-conjugated monosaccharides was monitored at *A*_400nm_ (*ε*_400nm, pH 12_ = 17,500 mol^−1^ L cm^−1^). Activity against arabinoxylan at 10 mg mL^−1^ was measured in McIlvaine buffer (pH 5.5) at 50 °C, for 10 min, using 15 µg mL^−1^ *Xac*Abf51 (263 nM) or 16 µg mL^−1^ (278 nM) *Tx*AbfD3 and the generation of arabinose from polysaccharides was determined by the 3,5-dinitrosalicylic acid (DNS) method [45]. To determine the kinetic properties of *Xac*Abf51 and *Tx*AbfD3, the reactions were performed in McIlvaine buffer (pH 5.5) at 50 °C, for 10 min, in the range from 7 µM to 14 mM of pNP-Ara*f* using 0.5 µg mL^−1^ (9 nM) *Xac*Abf51 or 0.1 µg mL^−1^ (2 nM) *Tx*AbfD3 and from 0.3 to 140 mg mL^−1^ of arabinan using 263 nM *Xac*Abf51 or 278 nM *Tx*AbfD3. The kinetic parameters were calculated by non-linear regression analysis of the Michaelis–Menten plot using the program OriginPro 8.1.

### Enzyme activity assays monitored by mass spectrometry

To estimate initial rates, the reactions were made in triplicate with 5 µL of oligosaccharide (XA^3^XX or XA^2+3^XX) in different concentrations (0.2–100 mM), 3 µL of McIlvaine buffer at pH 5.5, 1 µL of water and 1 µL of *Xac*Abf51 stock at 150 µg mL^−1^ (final enzyme concentration = 15 µg mL^−1^). After 5 min of incubation at 50 °C, 700 rpm, 40 µL of methanol was added to quench the reaction. Assays to compare the relative activity of *Xac*Abf51 and *Tx*AbfD3 on 2^3^,3^3^-di-α-l-arabinofuranosyl-xylotetraose (XA^2+3^XX) or 3^3^-α-l-arabinofuranosyl-xylotetraose (XA^3^XX) were performed incubating *Xac*Abf51 **(**15 µg mL^−1^) or *Tx*AbfD3 (15 µg mL^−1^) with 10 mM substrate, at pH 5.5, 50 °C, 700 rpm, 5 min (reaction volume = 10 µL) and quenched with 40 µL of methanol in triplicate.

The kinetic assays were monitored on a Waters Synapt HDMS, at *V* mode and ESI(+) with a spray voltage maintained at 3.0 kV and heated to 130 °C in the source. A total of 15 µL of the quenched reactions and 2 µL of 1 mM xylotriose (used as the internal standard) were added to 183 µL of water and injected into the mass spectrometer in scan mode (*m/z* 300–900) with direct infusion at a flow rate of 50 µL min^−1^. An internal standard with ionization similar to analytes (xylotriose) was used to increase the reliability of the method [46]. A calibration curve was made to determine the concentrations of the products of the enzymatic reaction. The kinetic parameters of the reactions (*k*_cat_, *K*_m_ and *V*_max_) were determined by non-linear regression analysis (Hill model) of the Michaelis–Menten plot using the software Origin8.1.

### Capillary zone electrophoresis

For capillary zone electrophoresis analysis, reactions were incubated for 60 min at 50 °C, pH 5.5, using 100 µg mL^−1^ *Xac*Abf51 and 10 mM AXOS, or 20 mg mL^−1^ arabinan, or 20 mg mL^−1^ arabinoxylan. Arabinose, xylobiose, xylotriose and xylotetraose (5 mM) and reactions without enzyme were used as standard references. Samples were heated at 95 °C, for 5 min, centrifuged and 30 µL of the supernatant was dried using Speed-Vac (Thermo Fisher Scientific, Waltham, MA). Reaction products were derivatized with 8-aminopyrene-1,3,6-trisulfonic acid (APTS) [47] by incubation with 20 µL 2.5 M citric acid, 8 µL 1 M sodium cyanoborohydride (in THF) and 1.5 µL 100 mM APTS (in 25% (v v^−1^) acetic acid), for 2 h at 75 °C. Labeled reactions (4 µL) or negative controls without enzyme (2 µL) were diluted to a final volume of 60 µL using run buffer (0.04 M potassium phosphate, pH 2.5) and injected into an uncoated fused-silica capillary of 75 µm internal diameter and 20 cm effective length (Beckman Coulter, Brea, CA), by application of 0.5 psi, for 5 s, using a P/ACE MDQ instrument configured with a laser-induced fluorescence detection system (Beckman Coulter, Brea, CA). Electrophoretic conditions were 20 kV/70–100 mA with reverse polarity at a controlled temperature of 25 °C. Carbohydrates labeled with APTS were excited at 488 nm and emission was collected through a 520-nm band pass filter.

### Preparation of enzyme cocktail from *T. reesei* Rut-C30

The strain *T. reesei* Rut-C30 was cultivated on Petri dishes containing potato dextrose agar (Difco, MI). After 6–10 days, spores were collected in spore solution (20% (v v^−1^) glycerol, 0.8% (m v^−1^)NaCl, 0.025% (v v^−1^) Tween 20), filtered through sterile cotton, quantified using a hemocytometer and frozen at − 80 °C for long-term storage.

Fermentations were performed using the BioFlo/CelliGen 115 system (Eppendorf, Hamburg, Germany) and water-jacketed 3.0-L vessels. The fermentation medium comprised of 5%  (m v^−1^) milled soybean hulls, 5% (v v^−1^) milk whey, 2% (m v^−1^) (NH_4_)_2_SO_4_ and 1 mL L^−1^ of J647 antifoam (Struktol, Hamburg, Germany) in the batch phase and milk whey with lactose concentration of 177 g L^−1^ were fed from 72 to 170 h at an average rate of 0.5 g L^−1^ h^−1^ total sugar. Aeration was maintained at 1.0 VVM compressed air, pH between 3.8 and 4.8 using 2 M phosphoric acid and 10% ammonia, and DO above 30% with an agitation cascade (400–950 rpm). The initial volume was 1 L, and the reactors were inoculated with 1:10 volume of 7-day-old shake flask preculture using the same media composition as the fermentation batch medium, spore concentration in the inoculum bottle was 2.5 × 10^7^ in 100 mL. Samples were withdrawn every 24 h, centrifuged at 21,000×*g* for 10 min and the supernatants stored at − 20 °C for analysis. Whole broth samples were adjusted to pH 5.0, frozen at − 20 °C and used for hydrolysis assays. Fermentations were terminated after 170 h when the feeding was stopped.

For quantifying protein, the sample was first diluted to a final concentration of 0.3–1.5 g L^−1^ in 50 mM Na citrate buffer, pH 5.0. A 200 μL sample was combined with 800 μL ice-cold acetone, mixed by inverting the tube several times and then maintained at − 20 °C for 1 h. The precipitated proteins were pelleted by centrifugation at 14,000×*g* and 4 °C for 5 min. The supernatant was removed and the pellet was air-dried for 5 min before resuspending in the original volume (200 μL) of buffer. The protein concentration was then quantified using the DC protein kit (BioRad, Hercules, CA) based on the method of Lowry [48] using bovine serum albumin as standard.

### Complementation assays

Delignified sugarcane bagasse was prepared using an alkaline pretreatment (130 °C, 30 min, 1.5% m v^−1^ NaOH), yielding a material composed by 58.6% cellulose, 22.1% hemicellulose, and 8.8% lignin. Enzymatic hydrolysis reactions were performed in samples of 1 mL containing 5% of dry biomass (50 mg) and 237.5 µg of enzyme cocktail, supplemented or not with 12.5 µg *Xac*Abf51, in buffer 50 mM sodium citrate, pH 5.5, with 0.02% sodium azide. The reactions were done in triplicate and incubated in a hybridization oven at 50 °C with agitation during 24 h. The enzyme cocktails used were Celluclast (Novozymes, Krogshoejvej, Denmark) and the whole broth from *T. reesei* RUT-C30, prepared as described above. Protein concentration was estimated by the Lowry method [48] using the DC protein kit (BioRad, Hercules, CA).

### Circular dichroism

CD spectra were acquired on a JASCO J-815 CD spectrometer (Jasco, Tokyo, Japan) controlled by a CDF-426S/15 Peltier temperature control system using a quartz cuvette with a 1-cm path length. The enzyme was prepared in phosphate buffer (20 mM sodium phosphate, 150 mM NaCl, pH 7.5) at a final concentration of 8 µM. All spectra were obtained at 20 °C in the range 195–260 nm with a bandwidth of 2 nm and a response time of 4 s nm^−1^. CD spectra were buffer subtracted and normalized to mean residue ellipticity. Thermal unfolding experiments were monitored at 220 nm in the temperature range 20–90 °C with a scan rate of 1 °C min^−1^. The melting temperature was determined according to the sigmoidal-Boltzmann fitting of the CD denaturation curve.

### Differential scanning calorimetry

Thermal stability was also analyzed by DSC using a VP-DSC device (Microcal, GE Healthcare, Northampton, MA). The enzyme was prepared in phosphate buffer (20 mM sodium phosphate, 150 mM NaCl, pH 7.5) at a final concentration of 2 mg mL^−1^. A temperature rate of 1 °C min^−1^ was used and the reversibility of protein denaturation was tested. Denaturation curves were buffer subtracted, concentration normalized and the resultant endotherms integrated following assignment of pre- and post-transition baselines.

### Dynamic light scattering

Size distribution of the purified enzyme in solution was evaluated using DLS. Measurements were acquired at 20 °C on a Malvern Zetasizer Nano ZS 90 (Model no. ZEN3690, Malvern, Worcestershire, UK) with a 633-nm laser, in a quartz cell with a scattering angle of 90°. The protein was analyzed at a concentration of 0.5 mg mL^−1^ in phosphate buffer (20 mM sodium phosphate, 150 mM NaCl, pH 7.5). An average of 20 runs was used to estimate the *R*_h_ through Stokes–Einstein equation.

### Analytical ultracentrifugation

Sedimentation velocity experiments were performed on a Beckman Optima XL-A analytical ultracentrifuge (Beckman Coulter, Indianapolis, IN) at 20 °C. Spectra were collected at both 220 and 280 nm. The protein was prepared in different concentrations ranging from 0.2 to 0.9 mg mL^−1^ in phosphate buffer (20 mM sodium phosphate, 150 mM NaCl, pH 7.5). AUC data were analyzed using the continuous sedimentation distribution method in the SEDFIT program [49]. The *s*_020,w_ value at infinite dilution was calculated by linear regression of *s*_20,w_ as a function of protein concentration.

### Small angle X-ray scattering

Small angle X-ray scattering measurements were performed at three different concentrations (2, 4 and 6 mg mL^−1^) in 20 mM Tris buffer, pH 7.5. Data were collected at SAXS2 beamline (LNLS, Campinas, Brazil), integrated using Fit2D [50] and analyzed using GNOM [51]. The molecular envelope was calculated from the experimental SAXS data using the program DAMMIN [52]. Ten runs of ab initio shape determination yielded highly similar models (normalized spatial discrepancy values < 1), which were then averaged using the package DAMAVER [53]. The theoretical scattering curves of crystallographic structures were calculated and compared with the experimental SAXS curves using the program CRYSOL [54]. The crystallographic structure was fitted into the SAXS molecular envelope using the program SUPCOMB [55].

### Protein crystallization, X-ray data collection and structure determination

*Xac*Abf51 (27 mg mL^−1^) crystallized by vapor diffusion method in sitting drops containing 17% (w v^−1^) polyethylene glycol 3350 and 0.2 M ammonium chloride. Crystals were cryoprotected using the reservoir solution added of 20% (v v^−1^) glycerol. Diffraction data were collected at the BL12-2 beamline from the Stanford Synchrotron Radiation Lightsource (Stanford, CA). Data were processed using XDS [56] and the structure was solved by molecular replacement method using the program MOLREP and the atomic coordinates of *Tx*AbfD3 (PDB ID: 2VRQ) as search model. Six chains were found in the asymmetric unit and the model was refined against electron density using COOT [57] and against X-ray data using phenix.refine [58] and REFMAC [59]. Final model was validated using MolProbity [60]. Data collection, processing and refinement statistics are summarized in Table 2.

**Table 2:** Data collection and refinement statistics of *Xac*Abf51 crystal structure

Data collection	
Space group	*P*2_1_
Cell dimensions	
*a*, *b*, *c* (Å)	91.34, 163.23, 114.42
β (º)	107.57
Resolution (Å)	48.74–1.91 (2.03–1.91)
*R*_meas_	0.113 (0.639)
*I*/σ*I*	9.74 (2.06)
Completeness (%)	95.1 (80.3)
Redundancy	3.4 (2.8)
CC ½	99.6 (72.9)

Refinement	
Resolution (Å)	48.74–1.91
No. of reflections	234,093
*R*_work_/*R*_free_	0.149/0.167
Protein residues	2964 (6 chains)
Ligands	6 GOL
Water	2384
*B*-factors (Å^2^)	
Average	24.64
Protein	24.00
Ligand	23.05
Water	30.75
R.m.s. deviations	
Bond lengths (Å)	0.0116
Bond angles (°)	1.5092
Ramachandran^a^	
Favored (%)	97
Allowed (%)	3
Outliers (%)	0
MolProbity score	1.00
PDB code	6D25

Values in parentheses are for highest resolution shell

^a^Ramachandran data were calculated using MolProbity

### Molecular dynamics simulation

Superimposition of *Xac*Abf51 crystal structure with that of *Tx*AbfD3 in complex with 3^2^-α-l-arabinofuranosyl-xylotriose (XA^3^X) was performed with PDBeFOLD [61] and the coordinates of the ligand positioned into the *Xac*Abf51 active site were transferred for the PDB file containing one trimer of *Xac*Abf51. An Ara*f* substitution was added to XA^3^X to generate XA^2+3^X and simulation systems using explicit solvent were created for energy-minimized trimeric structures of *Xac*Abf51 in complex with XA^2+3^X. Energy minimization and MD simulations were carried out using YAMBER3 force field with the program YASARA [62]. Long-range Coulomb interactions were included with a cutoff of 7.86 Å. The simulation box was defined at 15 Å around all atoms of the structure. Protonation was performed at pH 7. Cell neutralization was reached filling the box with water molecules (*d* = 0.997 g mL^−1^) and Na/Cl counter ions (0.9% m v^−1^) coupled with a short MD simulation for solvent relaxation. MD simulations were performed during 100 ns at 298 K, using a multiple time step of 2.0 fs for inter-molecular forces, 1.2 fs for intra-molecular forces, periodic boundary conditions and unconstrained bonds and angles. Root mean square deviations (RMSDs) were calculated for the whole system and Euclidean distances between enzyme and substrate atoms were measured through the trajectory in the three active sites of the trimer and the average value is presented in function of the simulation time.

### Phylogenetic analyses

The sequences of characterized GH51 enzymes present in the CAZY database, excluding redundant sequences (sequences from the same species with > 95% sequence identity) and synthetic constructs, were manually edited to include only the fragment corresponding to the (*β*/*α*)_8_ barrel, as predicted by the webserver SUPERFAMILY [63]. The edited sequences were aligned using the software MUSCLE, available at the EMBL-EBI webserver (https://www.ebi.ac.uk/Tools/msa/muscle/) [64]. The multiple sequence alignment was provided for the MEGA7 software to perform evolutionary analyses [65]. Initial tree(s) for the heuristic search were obtained automatically by applying Neighbor-Joining and BioNJ algorithms to a matrix of pairwise distances estimated using a JTT model, and then selecting the topology with superior log likelihood value. A discrete Gamma distribution was used to model evolutionary rate differences among sites [five categories (+*G*, parameter = 1.9905)]. The rate variation model allowed for some sites to be evolutionarily invariable ([+*I*], 1.20% sites). The analysis involved 72 amino acid sequences. All positions with less than 80% site coverage were eliminated. That is, fewer than 20% alignment gaps, missing data, and ambiguous bases were allowed at any position. There were a total of 292 positions in the final dataset. The confidence of tree topology was assessed using the Bootstrap analysis based on 1000 bootstrap replications [66].

## Abbreviations

Abf: α-l-arabinofuranosidase
Ara*f*: arabinofuranosyl
AUC: analytical ultracentrifugation
AXOS: arabinoxylooligosaccharides
*Bl*: *Bifidobacterium longum*
CD: circular dichroism
DSC: differential scanning calorimetry
DLS: dynamic light scattering
*Gs*: *Geobacillus stearothermophilus*
GH: glycoside hydrolase
*Hi*: *Humicola insolens*
MD: molecular dynamics
pNP: 4-nitrophenyl
NR: non-reducing end
R: reducing end
R_h_: hydrodynamic radius
*Rt*: *Ruminiclostridium thermocellum*
SAXS: small angle X-ray scattering
*Tx*: *Thermobacillus xylanilyticus*
*T*_m_: melting temperature
*Tm*: *Thermotoga maritima*
*Tp*: *Thermotoga petrophila*
*Xac*: *Xanthomonas axonopodis* pv. *citri*
*Xyl*p: xylopyranosyl
